# Temporal variation in oral microbiome composition of patients undergoing autologous hematopoietic cell transplantation with keratinocyte growth factor

**DOI:** 10.1186/s12866-023-03000-x

**Published:** 2023-09-13

**Authors:** Bruno Bohn, Miroslava Chalupova, Christopher Staley, Shernan Holtan, Joseph Maakaron, Veronika Bachanova, Najla El Jurdi

**Affiliations:** 1 Division of Epidemiology & Community Health, School of Public Health, University of Minnesota, 1300 S 2nd St, Minneapolis, MN 55455 USA; 2 Department of Stomatology, Faculty of Medicine and University Hospital in Pilsen, Charles University, Plzen, Czech Republic; 3 Department of Surgery, Medical School, University of Minnesota, Minneapolis, MN USA; 4 Division of Hematology, Oncology, and Transplantation, Department of Medicine, University of Minnesota, 420 Delaware Street SE, Minneapolis, MN 55455 USA

**Keywords:** Hematopoietic cell transplantation, Oral microbiome, Autologous

## Abstract

**Introduction:**

Autologous hematopoietic cell transplantation (AHCT) is a well-established treatment for lymphoma. Unintended effects of this therapy include oral mucositis (OM) and gastrointestinal toxicities, resulting in poor clinical outcomes. The gut microbiome has been previously linked to transplant toxicities among allogeneic recipients, but little is known about the effects of AHCT on the oral microbiome.

**Methods:**

Seven patients with non-Hodgkin or Hodgkin lymphoma undergoing AHCT with palifermin (keratinocyte growth factor) were included. Buccal swab samples were collected at baseline and 14- and 28-days post-treatment. Oral microbial communities were characterized with 16 S rRNA amplicon sequencing. Temporal trends in community composition, alpha diversity, and beta diversity were investigated.

**Results:**

A significant reduction in the relative abundance of the genera *Gemella* and *Actinomyces* were observed from baseline. No significant temporal differences in alpha diversity were observed. Significant changes in beta diversity were recorded.

**Conclusion:**

Results of this pilot study suggest treatment with AHCT and palifermin affects the oral microbiome, resulting in temporal shifts in oral microbial community composition. Future studies are warranted to confirm these trends and further investigate the effects of AHCT on the oral microbiome and how these shifts may affect health outcomes.

## Introduction

High-dose chemotherapy followed by autologous hematopoietic cell transplantation (AHCT) is a well-established curative treatment for chemotherapy-sensitive relapsed lymphoma. Patients undergoing AHCT experience regimen-related toxicities, including oral mucositis (OM) and gastrointestinal toxicities, leading to considerable morbidity and high readmission rates with significant impact on clinical and economic outcomes [1–6]. Conditioning regimens including chemotherapy and radiotherapy, directly and indirectly affect the mucosa of the digestive tract, leading to OM and other gastrointestinal toxicities, however the effects of those factors on temporal changes in the microbiome during the early transplant period remain largely unknown. Previous studies focused primarily on allogeneic recipients observed an association between the gut microbiome and transplant toxicities, however more evidence suggests the oral microbiome could play an important role in mediating AHCT toxicities and outcomes [1, 6–16].

Here we present a pilot study investigating the temporal changes in the oral microbiome of patients undergoing AHCT for lymphoma with the addition of palifermin, a keratinocyte growth factor that is approved to ameliorate OM and other gastrointestinal toxicities in the setting of AHCT [17–20]. Palifermin was added as a supportive care and quality improvement measure in our center for patients undergoing AHCT for lymphoma in an attempt to decrease regimen-related toxicities and improve outcomes [21]. We hypothesize that mucosal microbial dysbiosis and shifts in microbial community composition occur throughout the AHCT course and may linger without returning to pre-AHCT baseline after hematopoietic recovery from AHCT.

## Methods

### Study design and eligibly

Eligible patients were > 18 years of age, had a diagnosis of non-Hodgkin lymphoma (NHL) or Hodgkin lymphoma with a plan to undergo AHCT after conditioning with BEAM (busulfan, etoposide, cytosine arabinoside, melphalan). Patients received granulocyte colony-stimulating factor (G-CSF) mobilized peripheral blood stem cell graft. There were no early deaths, and all patients were followed up for at least 30 days after AHCT. All patients were on palifermin (60 mcg/kg/day), administered as an intravenous bolus injection for 3 consecutive days before and 3 consecutive days after myelotoxic therapy (two hours after stem cell graft infusion and the two subsequent days 1 and 2), for a total of 6 doses per FDA indication. Patients also received antimicrobial prophylaxis with levofloxacin (250 mg) orally daily and fluconazole (200 mg) orally daily. All patients started fluconazole on the day of start of conditioning (day − 6) and continued through day 60 post-AHCT. Levofloxacin was started on day − 1 through day 14 or later depending on neutrophil recovery (> 1000 cells/microL ) but discontinued before day 21 for all patients. Longitudinal buccal swab samples were collected with a 10-second rotating swab of the left and right buccal mucosa 5 times starting from the upper to the lower jaw with Puritan™ PurFlock™ Ultra Flocked swabs, placed directly in RNAlater vial. Samples were collected at least one hour after a meal. A total of three samples were collected from each patient: prior to AHCT (baseline), 14 days (D14) and 28 days (D28) from the day of hematopoietic cell infusion. The severity of oral mucositis and gastrointestinal toxicities were not prospectively collected for the study period. All participants provided informed consent to participating in this study. The study was approved by the Institutional Review Board at the University of Minnesota.

### DNA extraction and PCR amplification

Swab tips were cut using flame-sterilized scissors and placed into PowerBead tubes (QIAGEN, Hilden, Germany). Bacterial DNA was extracted with QIAGEN DNeasy® PowerSoil® Pro Kit on the automated QIAcube system (inhibitor removal technology protocol), following the manufacturer’s instructions. The V4 hypervariable region of the 16 S rRNA gene was amplified using the 515 F/806R primer set [22]. Polymerase chain reaction (PCR) amplification and dual-indexed, paired-end sequencing (300 nucleotides) were performed by the University of Minnesota Genomics Center (MN, USA) on the Illumina MiSeq platform (Illumina, Inc., San Diego, CA, USA) [23]. Negative controls (sterile water) were included in DNA extraction, PCR amplification, and sequencing and did not produce amplicons. Sequence data were deposited in the Sequence Read Archive under accession number SRP279100.

### Oral microbiome characterization

Sequencing data were processed and analyzed with mothur (v. 1.31.1) [24]. Sequences were trimmed to the first 170 nucleotides. Paired-ends were joined using fastq-join [25] and quality trimmed with 2 nucleotide primer mismatches, no ambiguous bases, homopolymers ≤ 8 nucleotides, and quality scores ≥ 35 over a 50-nucleotide window. For further processing, sequences were aligned against the SILVA database (v. 132) [26, 27]. Errors and chimeric sequences were removed using a 2% pre-cluster and UCHIME (v. 4.2.40) [28, 29]. Individual samples contained between 2 and 9575 high-quality reads. Operational taxonomic units (OTUs) were clustered at 99% similarity using the furthest-neighbor algorithm, and taxonomic classifications were made against the Ribosomal Database Project (v. 16) [30]. For calculations of diversity, samples were rarified to 1500 reads per sample (range of raw reads among samples included: 3013–17,892), resulting in the removal of five samples (baseline for patient #6 with 185 raw reads, D14 for patients #1, 4, 6, and 7 with 1003, 12, 30, and 31, respectively). Negative controls produced < 20 sequence reads and were removed from the dataset. Alpha diversity (i.e., within-sample diversity) was summarized with the Shannon Index and with the Chao1 Index. Beta diversity (i.e., between-sample diversity) was summarized with Bray-Curtis dissimilarity index. Good’s coverage estimates and diversity calculations were done using mothur.

### Statistical analyses

Differences in alpha diversity between the three time-points were investigated with analysis of variance (ANOVA) and pairwise differences with simple linear regression models. Beta diversity was explored with principal coordinate analysis (PCoA) and evaluated statistically using analysis of similarity (ANOSIM) [31]. Differences in alpha diversity and abundances of taxa were evaluated using ANOVA (parametric) or Kruskal-Wallis (non-parametric), depending on the distribution of the data. All statistics were evaluated at *α* = 0.05, with Bonferroni correction for multiple comparisons.

## Results

**Fig. 1:**
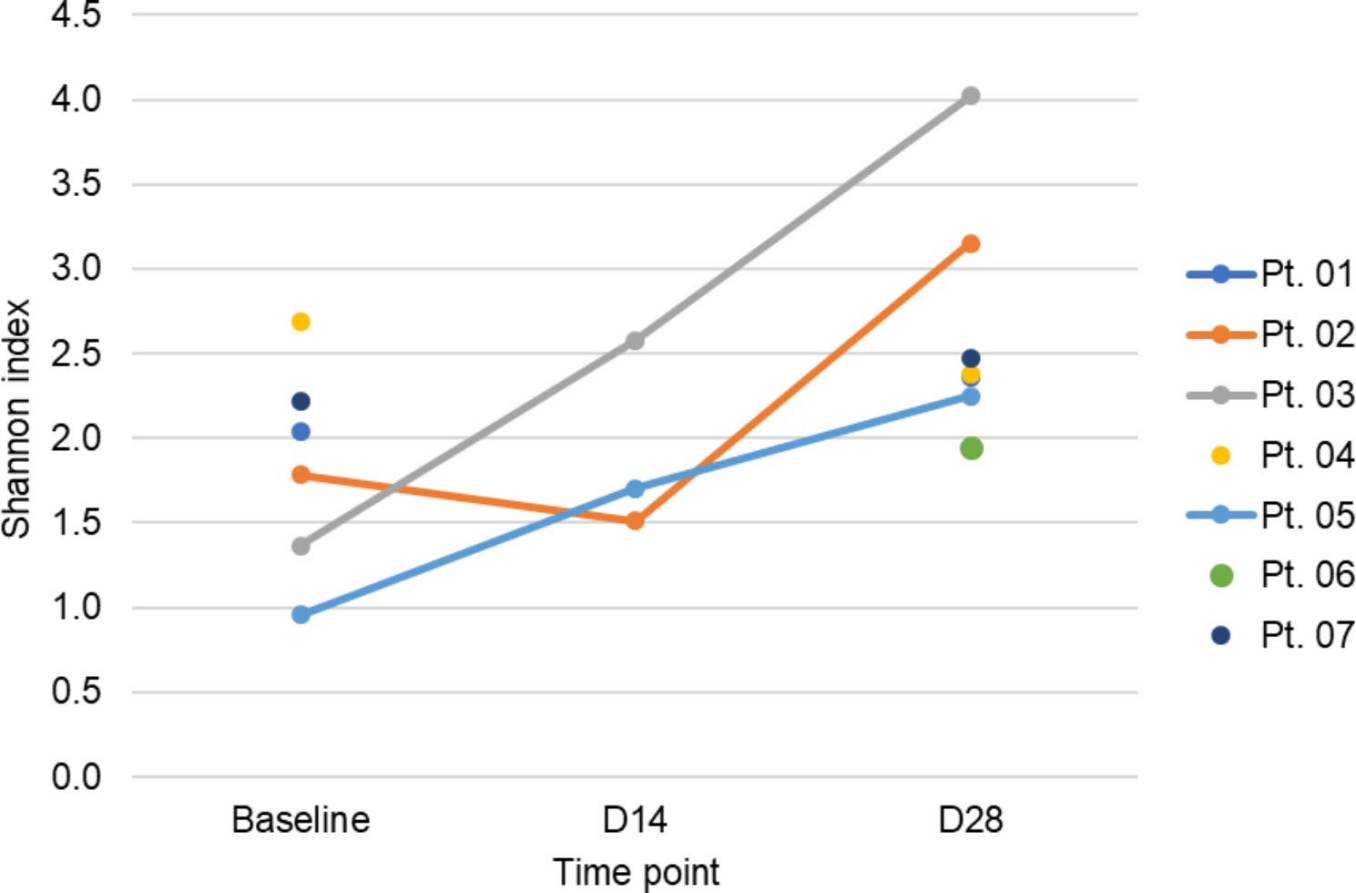
Spaghetti plot of Shannon indices for each patient

We enrolled 7 patients with median age of 63 years (range 26–75), 5 males and 2 females, one with a diagnosis of Hodgkin lymphoma and 6 with NHL (Table 1). Mean estimated Good’s coverage across all samples was 98.6 ± 1.6%. The mean Shannon Index was lowest at baseline (mean ± SD = 1.84 ± 0.62), followed by D14 (1.93 ± 0.57) and D28 (2.66 ± 0.70), but differences were not significant (ANOVA p-value = 0.093; Fig. 1). Similarly, the Chao1 index was also lowest at baseline (60.88 ± 32.74), followed by D14 (83.77 ± 51.98), and D28 (126.03 ± 136.45), but differences were not significant (ANOVA p-value = 0.493).

**Table 1:** Patient demographic and pre-transplant clinical characteristics for 7 US patients with non-Hodgkin or Hodgkin lymphoma undergoing autologous hematopoietic cell transplantation in 2020

	All (N = 7)
**Age at Transplant**: Median (Range)	63 (26–75)
**Sex**	
Male	5 (41%)
Female	2 (29%)
**Disease**	
Non-Hodgkin’s Lymphoma	6 (86%)
Hodgkin’s Lymphoma	1 (14%)
**Outcome**	
Alive	7 (100%)
**CD34**: Median (Range)	3.9 (3.5–10.6)
**KPS**: Median (Range)	90 (80–90)
**HCT-CI Score Group**	
0	1 (14%)
1–2	1 (14%)
>2	5 (72%)

Abbreviations: KPS, Karofsky Performance Score; HCT-CI, Hematopoietic Cell Transportation-specific Comorbidity Index; data collected at baseline pre-transplant

**Fig. 2:**
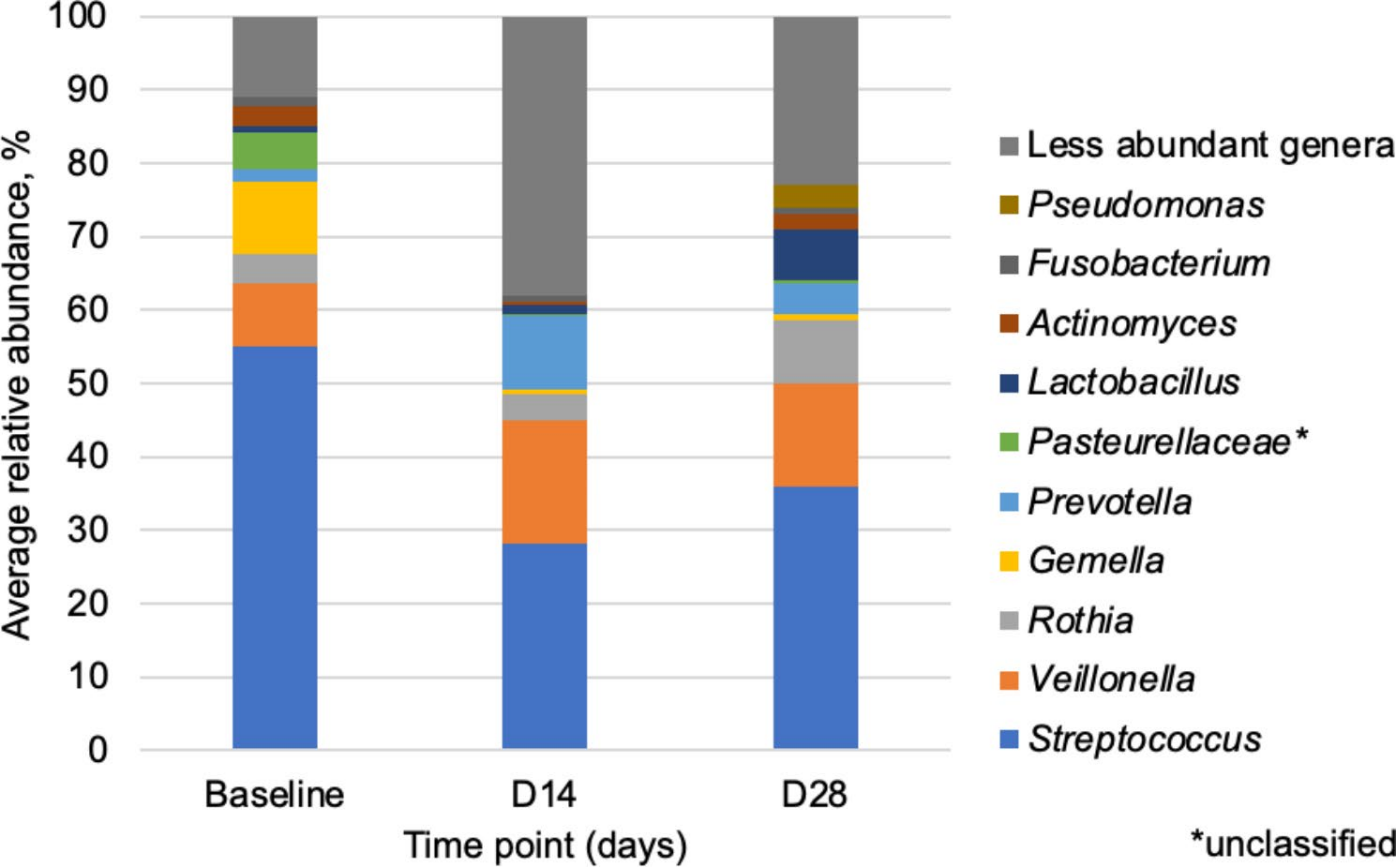
Average relative abundances of predominant genera at baseline and post-AHCT (14 and 28 days)

**Fig. 3:**
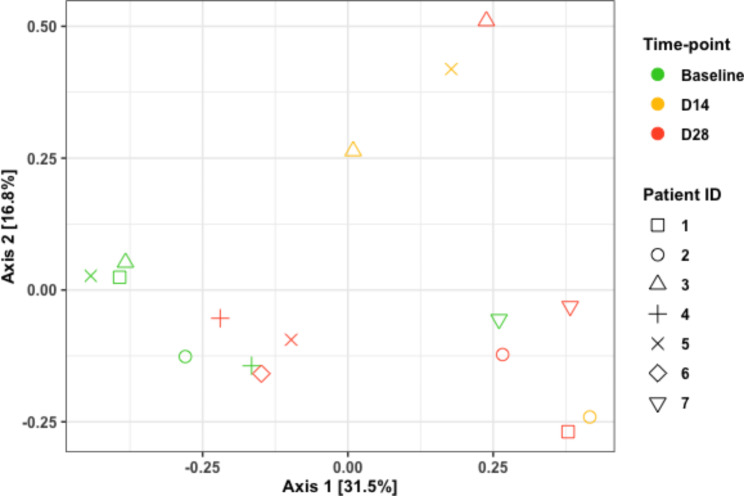
Principal Coordinates Analysis (PCoA) plot of Bray-Curtis Dissimilarity (Beta Diversity)

Overall shifts in microbial community composition were observed from baseline to post-treatment time points. The mean relative abundances across all time-points are shown in Fig. 2. We evaluated whether differences among the predominant taxa (those shown in Fig. 2) differed by time. Across all time-points, microbial communities were predominantly composed of the genera *Streptococcus* and *Veillonella*. For most participants (4/7), the relative abundance of *Streptococcus* decreased from baseline (mean = 55.0%) to D14 (mean = 28.3%), rebounding by D28 (mean = 36.0%) post-AHCT; these trends were not statistically significant (Kruskal-Wallis p = 0.255). Genera *Gemella* and *Actinomyces* were most altered by AHCT procedures. The relative abundance of *Gemella* was significantly greater at baseline (mean = 9.8%) compared to D14 (mean = 0.7%; p = 0.012) and remained low at D28 (mean = 0.9%; p = 0.041). The relative abundance of *Actinomyces* also decreased significantly from baseline (mean = 2.8%) to D14 (mean = 0.5%; p = 0.020), but recovered by D28 (mean = 2.0%, p > 0.05). Less abundant taxa were not interrogated due to data sparsity and a small number of samples that limited statistical analyses. An overall difference in community composition was observed across the three time points (ANOSIM R = 0.216, p = 0.028; Fig. 3); however, pairwise comparisons were not significant. A higher dissimilarity index was observed between the baseline and D14 samples (ANOSIM R = 0.556, p = 0.032; Bonferroni-adjusted α = 0.017) than between the baseline and D28 samples (ANOSIM R = 0.135, p = 0.086).

## Discussion

This pilot prospective study investigated the temporal changes in the oral microbiome of patients with lymphoma undergoing AHCT and treated with palifermin, added for mucosal cytoprotection. We observed that significant shifts in microbial community composition from baseline to D14 and D28 post-treatment, marked by reduction in *Gemella* and *Actinomyces* and a trend of decrease in *Streptococcus* from baseline to D14, followed by an increase by D28. While we noted no significant temporal differences in alpha (i.e., within-sample) diversity similar to previous reports, notable changes in beta (i.e., between-sample) diversity were recorded [1]. These results suggest that AHCT procedures with the use of high dose chemotherapy and antimicrobials are associated with temporal shifts in microbial community composition that persist beyond day 28 without returning to pre-treatment baselines despite the use of an oral cytoprotectant. Additionally, we hypothesize that the use of palifermin might explain the observed changes in beta diversity in this pilot study. We suspect that no significant differences in alpha diversity were observed due to a lack D14 samples with sufficient sequencing depth to include. Notably, all patients with a complete sample series tended to see an increase in Shannon index, although baseline and D28 Shannon indices were similar for the other three patients (Fig. 1).

Our observations have potentially unique clinical implications. *Streptococcus* and *Gemella* have been previously reported to dominate the oral microbial community in patients with breast cancer after chemotherapy exposure [32], while in patients undergoing AHCT for multiple myeloma, *Streptococcus* increased one week post-transplant followed by a subsequent decrease [1]. Our findings could potentially be explained by the prophylactic use of levofloxacin leading to decreased abundance of susceptible bacteria with effects that persist beyond the stop date and are organism dependent. It must be noted, however, that the impact of this medication on the gut microbiome has been previously characterized as mild [33], with its effects on the oral microbiome yet to be assessed. A study examining the oral microbiome in those with lymphoma or multiple myeloma undergoing AHCT noted an association between the decrease in Shannon diversity and the severity of oral mucositis [8]. Additionally, differences in our findings compared to other transplant cohorts [1] may be due to a different patient population, with different underlying diseases, treatment and conditioning regimen. This could be at least partially explained by the addition of palifermin in our cohort, as palifermin enhances the thickness of the oral mucosa epithelium and may impact niches of microorganisms. Oral dysbiosis is a reflection of multiple factors in this patient population including exposure to chemotherapy, antimicrobials and other medications that can have direct and indirect effects on the microbiome [34–37]. Our findings could support the hypothesis that peri-transplant OM associated changes in bacteriome are dominated by depletion of the most common oral commensals including *Streptococcus*, *Actinomyces* and *Gemella* [34]; although we have not objectively documented the severity of OM in this study, conditioning before AHCT is known to be clinically associated with some degree of OM in all patients. A previous study in patients undergoing AHCT for multiple myeloma noted that changes in oral microbial composition were more pronounced in those developing oral ulcers peri-transplant [12]. As noted in a prior study [28], compositional changes could not be attributed to the direct effects of the therapy, but rather reflected pro-inflammatory changes resembling other oral diseases. Changes in oral bacteria community composition and diversity have been linked to several oral and extra-oral outcomes, including infections, cardiometabolic disease, and recurring or new malignancies [38–41]. The associations between oral microbiome changes and risk of bacteremia, mucositis, and immune reconstruction remain uncharacterized, and our findings could assist in investigating this phenomenon and its effects on health.

This study has several noteworthy limitations. Firstly, due to the compositional nature of microbiome data, any observed changes in relative abundance of a given taxon are relative to the total abundance captured in the sample and thus may not be reflective of absolute changes in the microbiome or its biomass. Low microbial biomass in samples led to insufficient sequence results obtained, and the removal of 5 samples from some analyses. Due to this small sample size, the generalizability of our study and our ability to adjust for confounders was limited, further exacerbating the effects of confounding biases, as is the case with any observational study. However, due to the temporal nature of sampling, confounding due to non-time varying characteristics of the patients is less likely to be at play. Additionally, the direct effect of palifermin addition on the temporal changes in microbiome composition and diversity are not clear without a comparative cohort. Future investigations are warranted to further investigate our findings in a larger patient population, ideally comparing those changes to patients with similar disease and transplant characteristics not receiving palifermin. This pilot study serves as an important first step towards understanding the effects of AHCT with palifermin on the oral microbiome, uniquely reporting on longitudinal dynamic changes in oral microbiome composition in this patient population.

## Data Availability

Raw sequence data are available under SRA accession number SRP279100.
